# Epidemiological study of annual drowning cases in Fars province from April 2021 to March 2022: a cross-sectional study 

**Published:** 2022-12

**Authors:** Fariba Moradi Ardekani, Mehrab Sayadi, Mohsen Izadi

**Affiliations:** ^*a*^ Non-Communicable Diseases Research Center, Shiraz University of Medical Sciences, Shiraz, Iran.

## Abstract

**Background:**

The entry or aspiration of water or any other fluid into the lungs is called drowning. The World Health Organization (WHO) defines drowning as the process of breathing disorder from immersion in a liquid with the potential consequences of death, illness, or lack of complications. This study investigated the incidence of drowning so that by knowing its epidemiological distribution, more effective steps can be taken for prevention.

**Methods:**

This cross-sectional study was conducted to gather data from the Shiraz University of Medical Sciences (SUMS) database. Data were analyzed using descriptive statistics and Chi-square significant test by SPSS (Version 16.0. Chicago, SPSS Inc).

**Results:**

Eighty-seven individuals drowned in Fars province which according to the data obtained from SUMS, had 4,168,901 population in 2021. The incidence rate of drowning was 2.086 per 100,000 person-year. Their age varied from 1 to 70 years and 54.8% of them were under 20 years old. Of all drowning victims, 21.8% (n=19) were female and the rest were male. There was a significant difference in gender distribution (Chi-square statistic = 27.59, p less than 0.001). Most of them occurred in June and July months (n=24, 27.6 %). Most of the reported drownings (58.6%) were in warm seasons whereas 41.4% occurred in cold seasons. There was no significant difference between the rate of drownings in the warm and cold seasons (Chi-square = 2.58, p=0. 108). Most of the victims (55.2%, n=48) drowned in the pool.

**Conclusions:**

The results revealed that men were more at risk of drowning than women as well as younger people than the elderly. Given the evidence of uniform distribution of records throughout the months, most of them being in the pool, purposive prevention planning is necessary.

**Keywords:**

Drowning, Epidemiological, Prevention

